# Population-based age- and type-specific prevalence of human papillomavirus among non-vaccinated women aged 30 years and above in Germany

**DOI:** 10.1186/s12879-024-09827-7

**Published:** 2024-09-19

**Authors:** Linda A. Liang, Luana F. Tanaka, Kathrin Radde, Ulrike Bussas, Hans Ikenberg, Daniëlle A. M. Heideman, Chris J. L. M. Meijer, Maria Blettner, Stefanie J. Klug

**Affiliations:** 1 Chair of Epidemiology, TUM School of Medicine and Health, Technical University of Munich, Georg-Brauchle-Ring 56, 80992 Munich, Germany; 2 National Center for Tumor Diseases (NCT), NCT Heidelberg, a partnership between DKFZ and University Hospital Heidelberg, Heidelberg, Germany; 3 German Cancer Research Center, Division of Preventive Oncology, Heidelberg, Germany; 4 MVZ Cytomol, Frankfurt, Germany; 5 Amsterdam UMC Location Vrije Universiteit Amsterdam, Pathology, Amsterdam, The Netherlands; 6 Cancer Center Amsterdam, Imaging and Biomarkers, Amsterdam, The Netherlands; 7 Institute for Medical Biostatistics, Epidemiology, and Informatics, University Medical Centre of the Johannes Gutenberg-University Mainz, Mainz, Germany; 8 Present Address: Department of Pathology and Medical Biology, University Medical Center Groningen, University of Groningen, Groningen, The Netherlands; 9 Center for International Health, Ludwig-Maximilians-Universität München, Munich, Germany

**Keywords:** Human papillomavirus, Prevalence, Cervical cancer screening, Risk factors, Germany

## Abstract

**Background:**

A persisting high-risk human papillomavirus (HR-HPV) infection is causal for cervical cancer; however, there is limited population-based data on the prevalence of HPV infections in Germany. We assessed the age and type-specific HPV prevalence, and associated risk factors in HPV unvaccinated women aged 30 and above.

**Methods:**

The MARZY prospective population-based cohort study was conducted between 2005 and 2012 in Mainz and Mainz-Bingen, Germany. Eligible women were randomly recruited from population registries and invited for cervical cancer screening (*n* = 5,275). A study swab (liquid-based cytology) was taken and HPV testing was performed with GP5+/6 + polymerase chain reaction (PCR) followed by genotyping. We assessed HPV types as HR-HPV, ‘moderate’ risk and low-risk (LR-HPV). Logistic regression was performed to identify factors associated with HPV infection, stratified by HPV types.

**Results:**

2,520 women were screened with a valid PCR result. Overall HPV prevalence was 10.6% (*n* = 266), with 6.5% HR-HPV positive (*n* = 165), 1.5% ’moderate’ risk type (*n* = 38) and 3.3% LR-HPV type (*n* = 84) positive. 8.9% had a single infection (*n* = 225) and 1.6% had multiple types (*n* = 41). The most common HR-HPV types were 16, 56, 52 and 31 and LR-HPV 90 and 42. Of 187 HR-HPV infections detected (among 165 women), 55.1% (*n* = 103) were with HPV types not covered by available bivalent or quadrivalent HPV vaccines. About 23% (*n* = 43) were of types not covered by the nonavalent vaccine (HPV 35, 39, 51, 56, 59). The HR and LR-HPV prevalence were highest in the age group 30–34 years (HR 9.8%, ‘moderate’ risk 3.0% and LR 5.6%), decreasing with increasing age. HR-HPV prevalence in women with normal cytology was 5.5%. In women with a high-grade squamous intraepithelial lesion (HSIL), prevalence was 66.7%. Women currently not living with a partner and current smokers had increased chances of an HR-HPV infection.

**Conclusion:**

The overall population-based HPV prevalence was relatively high. An important share of prevalent HR-HPV infections constituted types not covered by current HPV vaccines. With the advent of HPV screening and younger vaccinated cohorts joining screening, HPV types should be monitored closely, also in older women who were not eligible for HPV vaccination.

**Supplementary Information:**

The online version contains supplementary material available at 10.1186/s12879-024-09827-7.

## Background

An infection with high-risk (HR) human papillomavirus (HPV) is a necessary cause for cervical cancer and contributes to almost all cervical cancer cases [1]. Currently, HR-HPV comprises types 16, 18, 31, 33, 35, 39, 45, 51, 52, 56, 58 and 59 ^2^, with HPV 16 and HPV 18 accounting for about 70% of all cervical cancers worldwide [3], while low-risk (LR) HPV types 6 and HPV 11 cause around 90% of genital warts. The persistence of specific HR-HPV types is associated with the development of high-grade cervical cancer lesions and cervical cancer [4–6]. Additional risk factors such as age, early sexual debut, parity, oral contraceptive use and tobacco smoking have also been observed to influence the onset of cervical cancer [6–9].

Given the role of HPV infection in cervical cancer development, effective primary and secondary prevention methods have been developed and continuously improved. Prophylactic HPV vaccination was first recommended by the World Health Organization (WHO) for girls aged 12 to 17 years in 2006 [10]. The WHO later revised the age group to target younger girls aged 9 to 14 years to ensure HPV vaccination prior to sexual debut. The first two HPV vaccines approved were Cervarix^®^ (bivalent) and Gardasil^®^ (quadrivalent), which cover the two most relevant HR-HPV types 16 and 18. The nonavalent vaccine Gardasil^®^9 covers additional HR-HPV types 31, 33, 45, 52 and 58. Both quadrivalent and nonavalent vaccines also protect against LR-HPV types 6 and 11 [11].

Prior to prophylactic vaccination against HPV and since the 1960s, cytology-based cervical cancer screening has been routinely offered in high income countries. In Europe, screening involved Pap smears obtained regularly between every one to five years and relied upon proper smear sampling, adequate assessment and quality assurance [12]. Despite its successes in reducing cervical cancer incidence and mortality, cytological screening is hampered by issues of poor sensitivity and reproducibility [13]. Recently, owing to the superior detection abilities and high negative predictive value of HPV testing compared to cytology [13], HPV testing has been implemented as a primary screening tool in several countries, such as the Netherlands and Australia [14]. In Germany, opportunistic screenings have been offered since 1971 and were based on annual cytological assessments with the Pap smear for women aged 20 years and above [15]. In 2020, an organised programme was implemented including HPV testing as a co-test alongside cytology at triennial intervals for women aged 35 and older. HPV testing includes target amplification methods such as GP5+/6 + polymerase chain reaction (PCR), capable of identifying HPV DNA and distinct genotypes. These methods can determine whether a screened woman has an HPV infection and whether this warrants further follow-up based on the oncogenicity of the HPV type [2].

The worldwide prevalence of overall HPV (HR and LR-HPV combined) in women with normal cytology is estimated at 11.7%, with major regional differences ranging from 9% in western Europe to 21.4% in eastern Europe [16]. Globally, Latin America (16.1%) and Sub-Sahara Africa (24.0%) have even higher HPV prevalence. These meta-analysis estimates are based on studies with women of all ages eligible for screening, including young women below 30 years of age in whom HPV infections are frequent [17]. Identifying the prevalence of HR-HPV in older women is important due to their ineligibility for HPV vaccination and higher risk of cervical cancer [18]. Previous studies reported HPV prevalences among women already attending routine screening [19] and few studies have focussed on women above the age of 29 years [20–25]. Among these older age groups, the HR-HPV prevalence in Germany ranged between 5 and 6% [20, 21, 25], and in women with normal cytology results, the prevalence ranged from 4 to 6% [20, 21, 25]. These studies, however, estimated prevalence from women attending screening opportunistically rather than from population-based samples. In opportunistic screening systems, uptake is known to be sub-optimal [12] and HPV prevalence in the general population appears to be substantially higher than in a screening population [6]. Results from other populations above 29 years including the United States, United Kingdom and Denmark demonstrated large variation in HR-HPV prevalence from less than 10% to up to 15–20% ^22–24^. Additionally, previous investigations of factors associated with HR-HPV infection were mostly conducted for all age groups, not specifically for women from age 30 years [23, 26–28].

This analysis aimed to estimate the population-based age and type-specific HR, moderate risk and LR-HPV prevalence in HPV unvaccinated women aged 30 years and above. In addition, the association of the different HPV types with socio-demographic characteristics and cytology results were investigated. These baseline estimates in unvaccinated women are relevant to determine and understand the mid- and long-term impact of HPV vaccination and screening efforts in the near future, especially as screening shifts towards primary HPV testing.

## Methods

### Study population

The MARZY study is a prospective population-based cohort study conducted between 2005 and 2012, investigating cervical cancer screening and HPV testing. The study design regarding invitation, screening and test accuracy is described in detail elsewhere [29, 30]. In brief, the study was conducted in two neighbouring regions in western Germany: Mainz, the capital of the state of Rhineland-Palatinate and the surrounding rural district of Mainz-Bingen. The study population (*n* = 5,275) aged between 30 and 65 years was randomly selected via population registries and invited via postal letter to participate in cervical cancer screening at a gynaecologist of their preference. The exclusion criteria were hysterectomy, pregnancy, childbirth in the past six months, temporary residence in the study area, history of cervical cancer, intellectual disability, transsexuality or employment at the study centre. HPV vaccinations in Germany were only approved in 2007 and for young girls, therefore, none of the study participants were eligible for vaccination.

### Study design

The present analyses were based on baseline data (2005–2007) of the cohort study. Participating women gave informed consent and completed a questionnaire prior to screening, documenting socio-demographic characteristics, history of participation in cervical cancer screening, and risk factors for cervical cancer, including smoking, oral contraceptive use, and hormone replacement therapy [29]. As described previously, the study swab was taken using an Ayres spatula and endocervical broom or a cytobrush if the transformation zone was not visible [30]. The swab was processed in PreservCyt^®^ solution for liquid-based cytology (ThinPrep^®^, Cytyc/Hologic^®^, Bedford, MA, USA) and was used for both cytological and hc2 HPV testing and processed centrally at a routine laboratory (CytoMol, Frankfurt, Germany).

### HPV testing and genotyping

The study swab was additionally used for HPV genotyping, and the testing procedures have been described in detail previously [30]. For these analyses, additional post-hoc HPV DNA testing was performed in a reference laboratory in The Netherlands (Department of Pathology, Amsterdam UMC, location Vrije Universiteit Amsterdam, Amsterdam) [31, 32]. For GP5+/6 + PCR HPV testing and genotyping, an aliquot of each PreservCyt^®^ sample was used for DNA extraction and 1/10 of the resulting DNA eluate was subjected to a human β-globin PCR reaction to verify the presence of sufficient amplifiable DNA. GP5+/6 + PCR was subsequently performed, followed by an enzyme immunoassay (EIA) using two cocktail probes: one for HR-HPV types 16, 18, 31, 33, 35, 39, 45, 51, 52, 56, 58, 59, 66, and 68 and another for HPV types 6, 11, 26, 30, 32, 34, 40, 42, 43, 44, 53, 54, 55, 57, 61, 64, 67, 69, 70, 71, 72, 73, 81, 82 (variants mm4 and is39), 83, 84, 85, 86, 89 (formerly cp6108), 90 (formerly jc9710). HPV genotyping of GP5+/6 + PCR-EIA positive samples was performed by reverse line blot (RLB) hybridisation of GP5+/6 + PCR products. Samples that were EIA positive but in which no genotypes could be detected by RLB were considered to contain uncharacterised types, referred to as HPV X.

### Classification of cytological results

The cytological results were originally classified according to the Munich Nomenclature II [33] and later, for analyses purposes, converted to the International Bethesda Classification for cytology [34] as: negative for intraepithelial lesion malignancy (NILM), atypical squamous cells of undetermined significance (ASC-US), low-grade intraepithelial lesion (LSIL) or high-grade intraepithelial lesion (HSIL) [33].

### Statistical analysis

In our analyses, HPV types were classified according to the International Agency for Research on Cancer (IARC) as HR with sufficient evidence (16, 18, 31, 33, 35, 39, 45, 51, 52, 56, 58 and 59) and limited evidence group 2 (26, 53, 66, 67, 68, 70, 73, 82), which was categorised as ‘moderate’ risk due to their potential for carcinogenicity [2]. All other HPV types were considered LR. Additionally, we classified HR-HPV types as being covered by the different nonavalent (16, 18, 31, 33, 45, 52 and 58), quadrivalent (16, 18) and bivalent (16, 18) vaccines. For the bivalent vaccine, we also considered HPV types 31, 33 and 45 for which cross-protection lasting at least seven years [35] or longer [36, 37] has been reported.

Age-specific overall HPV prevalence based on GP5+/6 + PCR test results with 95% confidence intervals (95% CI) using the Clopper-Pearson method were calculated. Additionally, the prevalence of individual HPV genotypes were determined. The prevalence of HR, ‘moderate’ risk and LR-HPV type groups were also reported stratified by cytology result. Associations between socio-demographic factors, other characteristics and HPV infection were assessed using univariable and multivariable logistic regression. Women who had infections with both HR and LR types or moderate risk types were categorised as HR. Analyses were stratified by HR-HPV, ‘moderate’ risk HPV, LR-HPV and HPV positive overall. A Cochran-Armitage trend test was performed in order to assess age trends.

Since the variables currently living with a partner and marital status were highly correlated, only the variable currently living with a partner was used in multivariable analyses. Since net household income and employment status were also highly correlated, only employment was included in the analysis. Smoking exposure as total cigarette pack-years was assessed. This was determined by the number of daily cigarettes reportedly smoked divided by 20 (the amount in a standard cigarette pack), multiplied by the duration of reported years of smoking [38]. The following variables were considered in multivariable regression due to their clinical relevance: age, living with a partner and smoking. Multiple imputation was applied for any missing data in both univariable and multivariable analyses, and results were pooled based on Rubin’s rule. All analyses were carried out using R (version 4.1.2, R Foundation for Statistical Computing, Vienna, Austria) and the R packages *survival* and *mice* were employed to carry out multiple imputed regression.

### Ethical considerations

The MARZY study was approved by the ethics committee of the state of Rhineland-Palatinate [Landesärztekammer Rheinland-Pfalz: 837.438.03 (4100)] and by the state government data protection office. The study was conducted following the guidelines of Good Epidemiological Practice (GEP).

## Results

In total, 5,275 women were eligible for inclusion in the study at baseline. Of those, 2,627 women (49.8%) participated in cervical cancer screening and received a study swab. 2,520 had a valid PCR test result and were included in the present analyses. The median age of the study population when receiving the baseline study swab was 46 years (95% CI 45, 46; range: 30–68 years). Few women aged above 65 years participated only after receiving a reminder but within the baseline study period.

Table 1 provides detailed information on the socio-demographic characteristics of the study population. Half of the screened women had reported ever smoking (49.0%), with 19.1% currently smoking. Among ever smokers 42.2% had moderate to heavy exposure to cigarette smoking (10+ total pack-years). Abnormal cytological findings (ASC-US+) were diagnosed in 3.7% of women. Overall, increasing age compared to the youngest age group (30–39) highlighted a lower likelihood of an HPV infection of any type and this likelihood decreased as age increased (Table 2). After adjustment of confounders, not living with a partner led to a 1.8-fold increase in HPV infection (adjusted odds ratio: aOR 1.78, 95% CI 1.28, 2.46) and high exposure to tobacco smoking (more than 20 total pack-years) led to a 1.6-fold increase (aOR 1.55, 95% CI 1.01, 2.39). Older age (60+ years) was also associated with a 63% reduced odds of HR-HPV infection (aOR 0.37, 95% CI 0.20, 0.68) compared to younger women. Women currently not living with a partner (aOR 1.72 95% CI 1.15, 2.56) and women who smoked 10 or more total pack-years (10–19 pack-years: aOR 1.94 95% CI 1.25, 3.03; 20+ pack-years: aOR 1.91, 95% CI 1.13, 3.24) had increased odds of HR-HPV infection.

**Table 1:** Socio-demographics of all MARZY study participants with a PCR result (*n* = 2,520), by HPV status

	HPV negative (*N* = 2,254)	HPV positive* (*N* = 266)	Total (*N* = 2,520)
	*n* (row %)	*n* (row %)	*n* (col %)**
**Age group (years)**			
30–39	584 (86.39%)	92 (13.61%)	676 (26.83%)
40–49	786 (89.73%)	90 (10.27%)	876 (34.76%)
50–59	520 (90.12%)	57 (9.88%)	577 (22.90%)
60+	364 (93.09%)	27 (6.91%)	391 (15.52%)
**Region**			
Mainz (urban)	980 (89.83%)	111 (10.17%)	1,091 (43.29%)
Mainz-Bingen (rural)	1,274 (89.15%)	155 (10.85%)	1,429 (56.71%)
**Nationality**			
German	2,033 (89.72%)	233 (10.28%)	2,266 (89.92%)
Non-German	221 (87.01%)	33 (12.99%)	254 (10.08%)
**Education**			
≤ 9 years	737 (89.01%)	91 (10.99%)	828 (32.86%)
10 years	704 (89.91%)	79 (10.09%)	783 (31.07%)
≥ 12 years	810 (89.40%)	96 (10.60%)	906 (35.95%)
Missing	3 (100.00%)	0 (0.00%)	3 (0.12%)
**Employment**			
Employed	1,379 (88.80%)	174 (11.20%)	1,553 (61.63%)
Not employed^a^	490 (87.81%)	68 (12.19%)	558 (22.14%)
Other^b^	337 (94.13%)	21 (5.87%)	358 (14.21%)
Missing	48 (94.12%)	3 (5.88%)	51 (2.02%)
**Household income (€ net/month)**			
≤ 1500	393 (84.88%)	70 (15.12%)	463 (18.37%)
1501–2500	642 (88.67%)	82 (11.33%)	724 (28.73%)
> 2500	704 (91.19%)	68 (8.81%)	772 (30.63%)
Missing	515 (91.80%)	46 (8.20%)	561 (22.26%)
**Marital status**			
Married, Divorced, Widowed	1,926 (90.08%)	212 (9.92%)	2,138 (84.84%)
Single	312 (86.19%)	50 (13.81%)	362 (14.37%)
Missing	16 (80.00%)	4 (20.00%)	20 (0.79%)
**Living with a partner**			
Yes	1,840 (90.64%)	190 (9.36%)	2,030 (80.56%)
No	293 (83.95%)	56 (16.05%)	349 (13.85%)
Missing	121 (85.82%)	20 (14.18%)	141 (5.60%)
**Parity**			
0	366 (86.32%)	58 (13.68%)	424 (16.83%)
1–2	1,419 (90.21%)	154 (9.79%)	1,573 (62.42%)
≥ 3	304 (90.21%)	33 (9.79%)	337 (13.37%)
Missing	165 (88.71%)	21 (11.29%)	186 (7.38%)
**Smoking status**			
Currently	411 (85.45%)	70 (14.55%)	481 (19.09%)
Past only	684 (90.60%)	71 (9.40%)	755 (29.96%)
Never	1,138 (90.25%)	123 (9.75%)	1,261 (50.04%)
Missing	21 (91.30%)	2 (8.70%)	23 (0.91%)
** If ever: Smoking duration**			
< 10 years	237 (89.43%)	28 (10.57%)	265 (21.05%)
10–19 years	359 (89.08%)	44 (10.92%)	403 (32.01%)
20–29 years	289 (87.05%)	43 (12.95%)	332 (26.37%)
≥ 30 years	153 (87.43%)	22 (12.57%)	175 (13.90%)
Missing	78 (92.86%)	6 (7.14%)	84 (6.67%)
** If ever: Smoking intensity (cig/day)**			
1–10	353 (88.92%)	44 (11.08%)	397 (31.53%)
10–19	421 (88.26%)	56 (11.74%)	477 (37.89%)
≥ 20	244 (87.46%)	35 (12.54%)	279 (22.16%)
Missing	98 (92.45%)	8 (7.55%)	106 (8.42%)
** If ever: Total pack-years smoking**			
< 10	540 (89.55%)	63 (10.45%)	603 (47.90%)
10–19	274 (87.54%)	39 (12.46%)	313 (24.86%)
≥ 20	187 (85.78%)	31 (14.22%)	218 (17.32%)
Missing	115 (92.00%)	10 (8.00%)	125 (9.93%)
**Oral contraception (OC) status**			
Currently	292 (83.91%)	56 (16.09%)	348 (13.81%)
Past only	1531 (90.16%)	167 (9.84%)	1,698 (67.38%)
Never	407 (90.65%)	42 (9.35%)	449 (17.82%)
Missing	24 (96.00%)	1 (4.00%)	25 (0.99%)
** If ever: OC duration**			
< 10 years	558 (89.57%)	65 (10.43%)	623 (30.08%)
10–19 years	751 (88.67%)	96 (11.33%)	847 (40.90%)
20–29 years	292 (88.22%)	39 (11.78%)	331 (15.98%)
≥ 30 years	53 (82.81%)	11 (17.19%)	64 (3.09%)
Missing	193 (93.69%)	13 (6.31%)	206 (9.95%)
**Hormone replacement therapy (HRT)**			
Currently	167 (92.27%)	14 (7.73%)	181 (7.18%)
Past only	272 (92.20%)	23 (7.80%)	295 (11.71%)
Never	1,753 (88.80%)	221 (11.20%)	1,974 (78.33%)
Missing	62 (88.57%)	8 (11.43%)	70 (2.78%)
** If ever: HRT duration**			
< 10 years	255 (92.06%)	22 (7.94%)	277 (50.73%)
10–19 years	93 (92.08%)	8 (7.92%)	101 (18.50%)
≥ 20 years	8 (100.00%)	0 (0.00%)	8 (1.47%)
Missing	145 (90.63%)	15 (9.38%)	160 (29.30%)
**Concurrent cytology abnormality**			
NILM	2,205 (90.82%)	223 (9.18%)	2,428 (96.35%)
ASC-US+	49 (53.26%)	43 (46.74%)	92 (3.65%)

* includes any type detected by GP5+/6 + PCR-EIA

** percentages for subcategories of smoking, oral contraception, and hormone replacement therapy calculated excluding women who have not reported their use

^a^ includes housewives, unemployed women

^b^ includes pensioners, students, women on maternity leave, ill women

HPV: human papillomavirus; NILM: Negative for Intraepithelial Lesion Malignancy; ASC-US+: atypical squamous cells of undetermined significance or worse

**Table 2:** Univariable and multivariable logistic regression analysis of HPV test results and potential associated risk factors, as detected by GP5+/6 + PCR-EIA and reverse line blotting

	High-Risk HPV vs. HPV Negative				‘Moderate’^a^ Risk HPV vs. HPV Negative				Low-Risk^b^ HPV vs. HPV Negative				HPV positive vs. HPV Negative			
	Univariable		Multivariable†		Univariable		Multivariable‡		Univariable		Multivariable‡		Univariable		Multivariable†	
	OR	95% CI	aOR	95% CI	OR	95% CI	aOR	95% CI	OR	95% CI	aOR	95% CI	OR	95% CI	aOR	95% CI
**Age**																
30–39	1	-	1	-	1	-	1	-	1	-	1	-	1	-	1	-
40–49	0.74	0.51, 1.08	0.69	0.47, 1.01	0.48	0.21, 1.06	0.48	0.22, 1.06	0.66	0.39, 1.12	0.66	0.39, 1.14	**0.73**	**0.53**,** 0.99**	**0.70**	**0.51**,** 0.96**
50–59	0.71	0.46, 1.09	0.64	0.41, 1.00	0.65	0.29, 1.49	0.65	0.28, 1.48	0.65	0.36, 1.20	0.65	0.35, 1.19	**0.70**	**0.49**,** 0.99**	**0.65**	**0.46**,** 0.93**
60+	**0.40**	**0.22**,** 0.72**	**0.37**	**0.20**,** 0.68**	0.32	0.09, 1.10	0.31	0.09, 1.06	0.62	0.31, 1.26	0.59	0.29, 1.19	**0.47**	**0.30**,** 0.74**	**0.44**	**0.28**,** 0.70**
*Trend test p-value*	*0.003*		*0.001*		*0.069*		*0.085*		*0.056*		*0.041*		*< 0.001*		*< 0.001*	
**Nationality**																
Non-German	1	-	1	-	1	-	1	-	1	-	1	-	1	-	1	-
German	0.91	0.55, 1.51	0.92	0.55, 1.54	0.59	0.25, 1.43	0.65	0.27, 1.59	0.66	0.35, 1.24	0.68	0.36, 1.27	0.77	0.52, 1.13	0.79	0.53, 1.17
**Study region**																
Urban (Mainz)	1	-	1	-	1	-	1	-	1	-	1	-	1	-	1	-
Rural (Mainz-Bingen)	1.22	0.88, 1.69	1.24	0.89, 1.72	0.55	0.29, 1.05	0.57	0.30, 1.10	1.02	0.66, 1.58	1.09	0.70, 1.70	1.07	0.83, 1.39	1.1	0.85, 1.43
**School education**																
< 12 years	1	-	1	-	1	-	1	-	1	-	1	-	1	-	1	-
≥ 12 years	1.05	0.75, 1.45	1.0	0.71, 1.41	1.45	0.76, 2.76	1.27	0.65, 2.48	0.89	0.56, 1.40	0.78	0.48, 1.26	1.00	0.77, 1.31	0.92	0.7, 1.22
**Employment**																
Employed	1	-	1	-	1	-	1	-	1	-	1	-	1	-	1	-
Not employed^c^	0.83	0.59, 1.16	1.07	0.74, 1.54	0.98	0.51, 1.91	1.25	0.61, 2.55	0.74	0.46, 1.18	0.83	0.49, 1.40	0.86	0.66, 1.13	1.05	0.78, 1.41
**Current living with a partner**																
Yes	1	-	1	-	1	-	1	-	1	-	1	-	1	-	1	-
No	**1.71**	**1.15**,** 2.54**	**1.72**	**1.15**,** 2.56**	1.95	0.91, 4.19	2.00	0.93, 4.30	**2.60**	**1.59**,** 4.27**	**2.63**	**1.60**,** 4.33**	**1.78**	**1.29**,** 2.45**	**1.78**	**1.28**,** 2.46**
**Parity**																
0	1	-	1	-	1	-	1	-	1	-	1	-	1	-	1	-
1–2	0.70	0.47, 1.03	0.86,	0.57, 1.29	**0.35**	**0.17**,** 0.72**	**0.42**	**0.19**,** 0.91**	0.71	0.41, 1.21	0.94	0.53, 1.67	**0.71**	**0.51**,** 0.97**	0.86	0.61, 1.20
≥ 3	0.68	0.39, 1.20	0.95,	0.53, 1.72	0.60	0.23, 1.59	0.80	0.29, 2.23	0.75	0.35, 1.59	1.09	0.49, 2.42	0.70	0.45, 1.10	0.93	0.58, 1.50
**Total pack-years smoked**																
None	1	-	1	-	1	-	1	-	1	-	1	-	1	-	1	-
< 10	1.19	0.79, 1.79	1.15	0.76, 1.73	0.59	0.24, 1.45	0.57	0.23, 1.41	1.09	0.64, 1.86	1.07	0.63, 1.85	1.07	0.78, 1.48	1.04	0.75, 1.44
10–19	**1.96**	**1.26**,** 3.04**	**1.94**	**1.25**,** 3.03**	0.57	0.17, 1.91	0.57	0.17, 1.93	1.00	0.49, 2.01	1.02	0.50, 2.08	1.29	0.88, 1.90	1.29	0.87, 1.90
20+	**1.84**	**1.10**,** 3.06**	**1.91**	**1.13**,** 3.24**	1.34	0.50, 3.57	1.44	0.52, 3.97	1.60	0.81, 3.18	1.58	0.78, 3.19	1.51	0.99, 2.30	**1.55**	**1.01**,** 2.39**
**Oral contraceptive duration**																
None	1	-	1	-	1	-	1	-	1	-	1	-	1	-	1	-
< 10 years	1.22	0.72, 2.05	1.03	0.60, 1.77	1.98	0.63, 6.26	1.71	0.53, 5.53	0.86	0.42, 1.78	0.82	0.39, 1.74	1.14	0.76, 1.72	1.00	0.66 1.53
10–19 years	1.42	0.88, 2.30	1.12	0.68, 1.86	2.01	0.66, 6.09	1.69	0.54, 5.26	1.29	0.68, 2.43	1.20	0.62, 2.33	1.25	0.86, 1.83	1.06	0.71, 1.58
20–29 years	1.32	0.73, 2.38	1.25	0.69, 2.29	1.74	0.46, 6.53	1.87	0.49, 7.10	1.38	0.65, 2.93	1.57	0.73, 3.37	1.31	0.83, 2.07	1.31	0.82, 2.09
30+ years	**2.46**	**1.06**,** 5.73**	2.41	1.00, 5.79	3.52	0.63, 19.64	3.83	0.66, 22.34	1.53	0.43, 5.47	1.89	0.51, 6.99	1.98	0.97, 4.07	2.08	0.99, 4.38
**Hormone replacement therapy**																
Never	1	-	1	-	1	-	1	-	1	-	1	-	1	-	1	-
Ever	**0.53**	**0.32**,** 0.87**	0.66	0.37, 1.17	0.27	0.07, 1.10	0.29	0.06, 1.35	1.23	0.73, 2.07	1.82	0.94, 3.54	**0.68**	**0.47**,** 0.97**	0.85	0.56, 1.31
**Cytological result**																
NILM	1	-	NA				NA				NA				NA	
ASC-US+	**8.70**	**5.46**,** 13.87**			**5.22**	**2.13 12.83**			**3.88**	**1.93**,** 7.78**			**8.68**	**5.63**,** 13.37**		

† adjusted for all age, living with a partner and smoking

‡ adjusted for age and living with a partner (smoking is not considered due to the lack of evidence in literature and no associations observed in sensitivity analyses)

^a^ includes types: 26, 53, 66, 67, 68, 70, 73, 82

^b^ includes types: 6, 11, 30, 32, 40, 42, 43, 54, 55, 71, 72, 81, 83, 86, 89, 90

^c^ includes housewives, pensioners, students, women on maternity leave, ill women

OR: Odds Ratio; CI: Confidence interval; aOR: adjusted Odds Ratio; NILM: Negative for Intraepithelial Lesion Malignancy; ASC-US+: atypical squamous cells of undetermined significance or worse; NA: not applicable

### HPV prevalence

The overall prevalence of any HPV infection was 10.6% (*n* = 266, 95% CI 9.4, 11.8). Of the 266 HPV positive women, the majority (84.6%) had a single infection (*n* = 225, 95% CI 79.7, 88.7). After counting all HPV infections separately (Table 3), regardless if they occurred in women as a single or multiple infections, HR-HPV prevalence was 6.5% (*n* = 165, 95% CI 5.6, 7.6), IARC classified types with limited evidence or ‘moderate’ risk was 1.5% (*n* = 38, 95% CI 1.1, 2.1) and LR-HPV prevalence was 3.3% (*n* = 84, 95% CI 2.7, 4.1).

**Table 3:** Type-specific HPV distribution detected by PCR GP5+/6 + and reverse line blot assay, total sample (*n* = 2,520), all HPV positive women (*n* = 266), and all HR-HPV positive women (*n* = 165), stratified by subgroups of HR-HPV, IARC group 2 (‘moderate’) HPV and LR-HPV types

	*n*	% (total sample)	95% CI (total sample)	% (of HPV positive)	95% CI (of HPV positive)	% (within subgroup)	95% CI (within subgroup)
**High-risk-HPV**	165^1^	6.5	5.6, 7.6			100.0	
HPV 16 ^b, q, n^	71	2.8	2.2, 3.5	26.7	21.5, 32.4	43.0	35.4, 51.0
HPV 18 ^b, q, n^	13	0.5	0.3, 0.9	4.9	2.6, 8.2	7.9	4.3, 13.1
HPV 31 ^n (b)^	15	0.6	0.3, 1.0	5.6	3.2, 9.1	9.1	5.2, 14.6
HPV 33 ^n (b)^	11	0.4	0.2, 0.8	4.1	2.1, 7.3	6.7	3.4, 11.6
HPV 35	4	0.2	0.0, 0.4	1.5	0.4, 3.8	2.4	0.7, 6.1
HPV 39	9	0.4	0.2, 0.7	3.4	1.6, 6.3	5.5	2.5, 10.1
HPV 45 ^n (b)^	13	0.5	0.4, 1.0	4.9	2.6, 8.2	7.9	4.3, 13.1
HPV 51	4	0.2	0.0, 0.4	1.5	0.4, 3.8	2.4	0.7, 6.1
HPV 52 ^n^	16	0.6	0.4, 1.0	6.0	3.5, 9.6	9.7	5.6, 15.3
HPV 56	23	0.9	0.6, 1.4	8.6	5.6, 12.7	13.9	9.0, 20.2
HPV 58 ^n^	5	0.2	0.1, 0.5	1.9	0.6, 4.3	3.0	1.0, 6.9
HPV 59	3	0.1	0.0, 0.3	1.1	0.2, 3.3	1.8	0.4, 5.2
Sub-total infections	187						
**IARC types with limited evidence group 2 ‘moderate’ HPV**	38^2^	1.5	1.1, 2.1			100.0	
HPV 26	0	-	-	-	-	-	-
HPV 53	0	-	-	-	-	-	-
HPV 66	19	0.8	0.5, 1.2	7.1	4.4, 10.9	50.0	33.4, 66.6
HPV 67	3	0.1	0.0, 0.3	1.1	0.2, 3.3	7.9	1.7, 21.4
HPV 68	1	0.0	*	0.4	0, 2.1	2.6	0.1, 13.8
HPV 70	11	0.4	0.2, 0.8	4.1	2.1, 7.3	28.9	15.4, 45.9
HPV 73	4	0.2	0.0, 0.4	1.5	0.4, 3.8	10.5	2.9, 24.8
HPV 82	1	0.0	*	0.4	0, 2.1	2.6	0.1, 13.8
Sub-total infections	39						
**Low-risk HPV**	84^3^	3.3	2.7, 4.1			100.0	
HPV 6 ^b, q,n^	5	0.2	0.1, 0.5	1.9	0.6, 4.3	6.0	2.0, 13.3
HPV 11 ^b, q,n^	2	0.1	*	0.8	0.1, 2.7	2.4	0.3, 8.3
HPV 30	1	0.0	*	0.4	0, 2.1	1.2	0.0, 6.5
HPV 32	1	0.0	*	0.4	0, 2.1	1.2	0.0, 6.5
HPV 40	6	0.2	0.1, 0.5	2.3	0.8, 4.8	7.1	2.7, 14.9
HPV 42	24	1.0	0.6, 1.4	9.0	5.9, 13.1	28.6	19.2, 39.5
HPV 43	7	0.3		2.6	1.1, 5.3	8.3	3.4, 16.4
HPV 54	4	0.2	0.0, 0.4	1.5	0.4, 3.8	4.8	1.3, 11.7
HPV 55	3	0.1	0.0, 0.3	1.1	0.2, 3.3	3.6	0.7, 10.1
HPV 71	0	0.0	-	-	-	-	-
HPV 72	2	0.1	*	0.8	0.1, 2.7	2.4	0.3, 8.3
HPV 81	1	0.0	*	0.4	0, 2.1	1.2	0.0, 6.5
HPV 83	6	0.2	0.1, 0.5	2.3	0.8, 4.8	7.1	2.7, 14.9
HPV 86	1	0.0	*	0.4	0, 2.1	1.2	0.0, 6.5
HPV 89 (cp6108)	1	0.0	*	0.4	0, 2.1	1.2	0.0, 6.5
HPV 90 (jc9710)	28	1.1	0.7, 1.6	10.5	7.1, 14.9	33.3	23.4, 44.5
Sub-total infections	92						
Unspecified types HPV X	13	0.5	0.3, 0.9	4.9	2.6, 8.2		

^1^ Refers to the number of women with HR-HPV, regardless of single or multiple infections

^2^ Refers to the number of women with IARC types with limited evidence, regardless of single or multiple infections

^3^ Refers to the number of women with Low-risk HPV, regardless of single or multiple infections

^b, q, n^ covered by the bivalent (Cervarix^®^), quadrivalent (Gardasil^®^) and nonavalent (Gardasil^®^9) vaccines

^n (b)^ covered by the nonavalent (Gardasil^®^9) and potentially cross-protective by the bivalent (Cervarix^®^) vaccines (based on data with ≥ 7 years follow-up data [35–37])

^n^ covered by the nonavalent (Gardasil^®^9) vaccine

* no confidence interval due to small estimate

HPV: human papillomavirus; IARC: International Agency for Research on Cancer

### Type-specific HPV prevalence

Among all 2,520 women with a PCR result, HPV 16 was the most frequent type (2.8%) (Table 3). We observed 187 infections with HR-HPV types among 165 women. Among HR-HPV types, HPV 16 contributed to almost half the HR types detected (43.0%, 95% CI 35.4, 51.0), followed by HPV 56 (13.9%, 95% CI 9.0, 20.2), HPV 52 (9.7%, 95% CI 5.6, 15.3) and HPV 31 (9.1%, 95% CI 5.2, 14.6). About half (55.1%, *n* = 103) of all the 187 HR-HPV infections were with types not covered by the quadrivalent vaccine or the bivalent vaccine, already taking into account cross protection of HPV types 31, 33, and 45 [35–37]. A quarter (23%, *n* = 43) of detected infections in our population were not covered by the nonavalent vaccine (Supplements Table S1).

As for the HPV types of ‘moderate risk’, HPV 66 ranked first among its subgroup and accounted for half of these infections (50.0%, 95% CI 33.4, 66.6), corresponding to 0.8% (95% CI 0.5, 1.2%) of all women included and 7.1% (95% CI 4.4, 10.9) of all HPV types detected (Table 3). Among the women positive for LR-HPV, HPV 90 and HPV 42 were the most common types (33.3%, 96% CI 23.4, 44.5 and 28.6%, 95% CI 19.2, 39.5 respectively). Both types each represented approximately 1% of all HPV types detected in all HPV-tested women. Only five women were positive for HPV 6 (0.2%, 95% CI 0.1, 0.5%) and 2 for HPV 11 (0.1%).

### Age-specific HPV prevalence

**Fig. 1:**
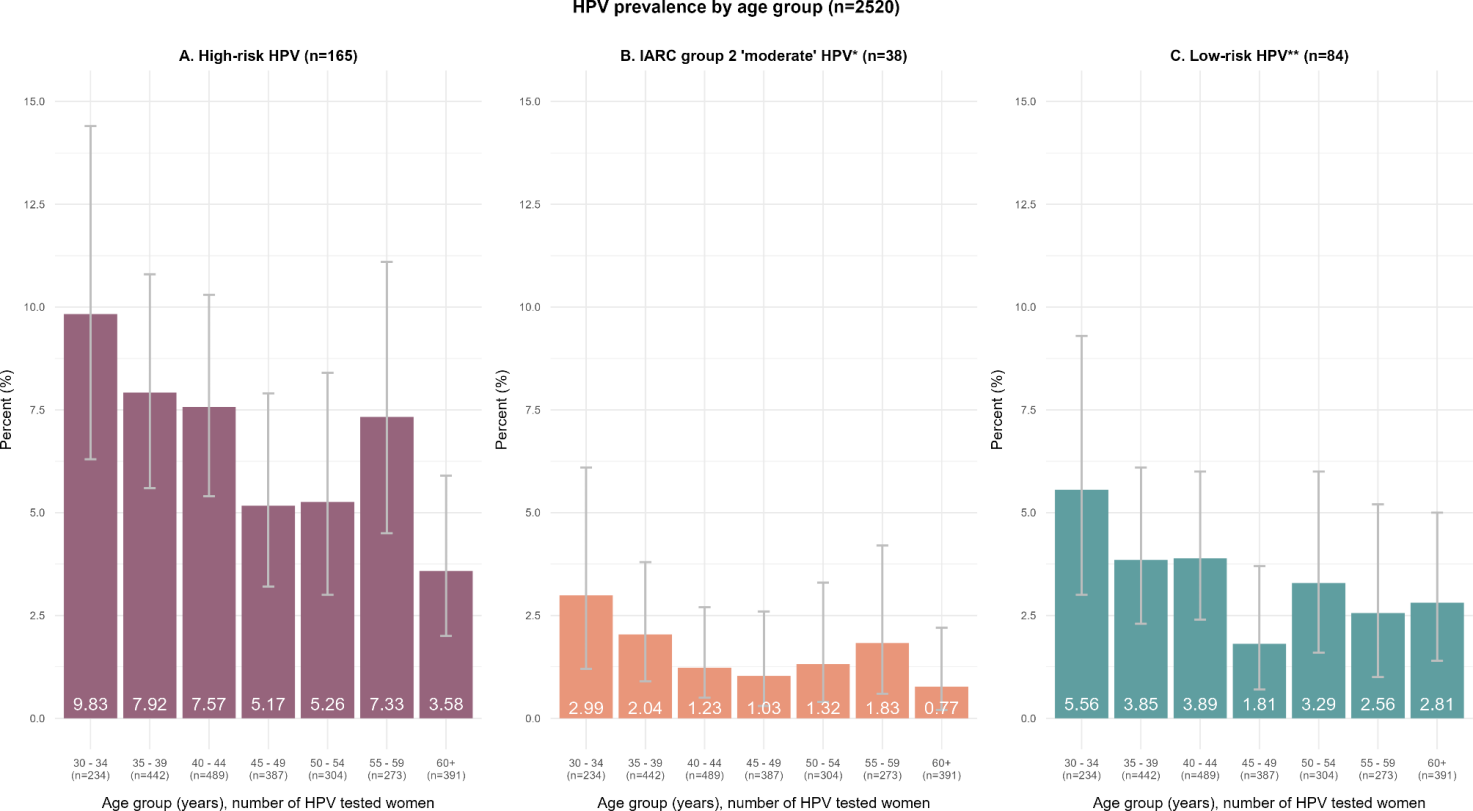
HPV prevalence by 5-year age groups of the total sample of 2,520 women, stratified by high-risk (HR) HPV (**A**), IARC group 2 ‘moderate’ HPV (**B**) and low-risk (LR) HPV (**C**). * includes types: 26, 53, 66, 67, 68, 70, 73, 82. ** includes types: 6, 11, 30, 32, 40, 42, 43, 54, 55, 71, 72, 81, 83, 86, 89, 90

Age-specific HR-HPV, ‘moderate’ risk and LR-HPV prevalence are shown in Fig. 1. HR-HPV prevalence decreased from 9.8% (95% CI 6.3, 14.4%) in the youngest age group (30–34 years) to 3.6% (95% CI 2.0, 5.9%) in the oldest age group (60+ years), including a slight increase of prevalence between ages 55–59 years (Fig. 1A). Similar decreasing patterns of prevalence with increasing age were observed for ‘moderate’ types (Fig. 1B) and LR-HPV (Fig. 1C). A statistically significant inverse linear trend was found between age and the prevalence of HR-HPV infection (*p* = 0.003; Table 2).

### Prevalence of HPV by cytological findings

The large majority (96.3%) of women in the study population had normal cytological findings (NILM; Table 1). Among these women with normal cytology, 9.2% were positive for any HPV type, 5.5% for a HR type, 1.3% were positive for a ‘moderate’ risk type and 3.1% were positive for any LR-HPV (Table 4). In women with any borderline to low-grade cytological abnormality (ASC-US/LSIL), HR-HPV was detected in 31.4% (*n* = 27) and LR-HPV in 11.6% (*n* = 10). Two-thirds of the women with HSIL (66.7%; *n* = 4) had an HR-HPV infection. Single HPV infections were observed in 7.7% of NILM results (*n* = 188), 38.4% of ASC-US or LSIL results (*n* = 33) and 66.7% in HSIL results (*n* = 4). 7.0% of abnormal and low-grade results had multiple HPV types (*n* = 6).

**Table 4:** Prevalence of HPV infection (PCR GP5+/6 + and reverse line blot assay) by cytological findings (total sample *n* = 2520)

	NILM (Pap I/II)		ASC-US/LSIL (Pap IIw/IIk/III/IIID)		HSIL (Pap IV)		TOTAL	
	*n* = 2,428		*n* = 86		*n* = 6		*n* = 2,520	
	*n*	%	*n*	%	*n*	%	*n*	%
HPV positive (any type)	223	9.2	39	45.4	4	66.7	266	10.6
HPV negative	2,205	90.8	47	54.7	2	33.3	2,254	89.4
HR-HPV*^a^ types	134	5.5	27	31.4	4	66.7	165	6.5
Only HR-HPV (no other types)	111	4.6	23	26.7	4	66.7	138	5.5
IARC limited evidence ‘moderate’ risk *^b^ types	32	1.3	6	7.0	0	0	38	1.5
Only limited evidence (no other types)	21	0.9	3	3.5	0	0	24	1.0
LR-HPV*^c^ types	74	3.1	10	11.6	0	0	84	3.3
Only LR-HPV (no other types)	56	2.3	8	9.3	0	0	64	2.5
Single HPV Type	188	7.7	33	38.4	4	66.7	225	8.9
Multiple HPV Types	35	1.4	6	7.0	0	0	41	1.6
Detected with both HR-HPV & ‘moderate’ risk types	31	1.3	5	5.8	0	0	36	1.4

* including women with multiple infections

^a^ types include: 16, 18, 31, 33, 35, 39, 45, 51, 52, 56, 58, 59

^b^ types include: 26, 53, 66, 67, 68, 70, 73, 82

^c^ types include: 6, 11, 30, 32, 40, 42, 43, 54, 55, 71, 72, 81, 83, 86, 89, 90

NILM: Negative for Intraepithelial Lesion or Malignancy; ASC-US: Atypical squamous cells of undetermined significance; LSIL: Low grade squamous intraepithelial lesion; HSIL: High grade squamous intraepithelial lesion; HPV: human papillomavirus; HR-HPV: high-risk HPV; LR-HPV: low-risk HPV, IARC: International Agency for Research on Cancer

## Discussion

Our study investigated age and type-specific HPV prevalence as well as socio-demographic and risk factors in a population-based sample of HPV unvaccinated women aged 30 and above in Germany. In the total population, overall HPV positivity was 10.6% and HR-HPV prevalence was 6.5%, highest among younger women and consistently decreasing with increasing age. HR-HPV prevalence in women with normal cytological results was 5.5%. HPV prevalence of ‘moderate’ types was 1.5% and 3.3% had LR-HPV infections. The most common HR-HPV types detected were 16, 18, 31, 45, 52 and 56. Interestingly, of the 187 HR-HPV infections observed, 55% comprised HPV types not covered by bivalent or quadrivalent vaccines, and 23% were not covered by nonavalent vaccines. Our findings highlight the need to monitor the prevalence of these non-vaccine covered but HR-HPV genotypes over time to assess potential consequences for screening and vaccination strategies.

A global meta-analysis estimated an overall HPV prevalence in women with normal cytology of 9.0% (95% CI 8.8, 9.2) for western Europe [16], comparable to our results (9.5%). These western European estimates included two previous German studies that were not population-based by design, as well as data from Belgium, France and the Netherlands [16], which are countries with fairly similar cervical cancer incidence [39]. We report a HR-HPV prevalence of 6.5% using GP5+/6 + PCR in our sample of women aged 30 years and older with a median age of 46 years (95% CI 45, 46), which included previously unscreened women [29]. Other studies reported much higher HR-HPV prevalence in women above 30 years, ranging from approximately 15% in Denmark [24] and up to 28% in the United States [23]. It is possible that selection of study sample, different HPV assays, categorisation of more HPV types as HR and use of self-collected vaginal smears contribute to these differences [23, 24]. Among women attending opportunistic screening in Germany, HR-HPV prevalence (6.4% overall and 3.7% among women with normal cytology) were comparable to our findings [20].

### Type-specific prevalence

In our study, HPV 16 was by far the most frequent HR-HPV type, affecting 2.8% of all women in the study sample, with a proportion of 43% of HR-HPV positive women. This is in agreement with previous studies in women aged over 30 years [20–22]. In contrast, HPV 18, considered to be the second most common HR-HPV type worldwide and third most common in Europe after HPV 31 [16], only ranked fifth in our study with a prevalence of 0.5% (7.9% of all HR-HPV infections), following HPV types 16, 56, 52 and 31. Studies from Germany [21] and other European countries, including Denmark and Netherlands, also identified genotypes other than HPV 18, such as HPV 52, 51, 31, 33, as the most frequently observed types [24, 40, 41].

### Impact of HPV vaccination status on screening

Interestingly, we observed that 23% of all HR-HPV infections detected were of types not covered by any of the available vaccines. This information is important, firstly because our results provide baseline evidence of the HR-HPV types prevalent in the HPV non-vaccinated population, which impacts current and future screening and disease management policies [18]. It highlights the necessity to continue screening efforts, particularly since women who are immunised against HPV, are not fully protected against all the HR-HPV types associated with cervical cancer, even considering cross-protection effects [35–37]. Second, although younger cohorts are eligible for HPV vaccination, HPV vaccination coverage in Germany and in other countries worldwide is lagging. In 2020, only 54.1% of 18 year old women in Germany were fully immunised [42]. This underscores the importance of maximising both primary and secondary prevention measures by improving vaccination rates and screening uptake, also in high income countries such as Germany.

### Benefits of HPV type monitoring

We also report a strong association between abnormal cytological diagnosis and HPV infection consistent with the literature [1, 16]. This association was observed in a large meta-analysis of HPV-positive women worldwide, showing particular HR-HPV types such as HPV 16 to be common contributors to HPV infections among women with invasive cervical cancers [27]. Assessing both HPV and cytological status as triage is an important step for narrowing risk and guiding management [18].

As for ‘moderate’ risk and LR-HPV types, HPV 90 (1.1%), HPV 42 (1.0%) and HPV 66 (0.8%) were the three most commonly found HPV types in this study. However, HPV prevalence and distribution varies by population and region. In a population-based Danish study [24], HPV 6 (1.6%) and HPV 74 (1.4%) were most prevalent among the LR-HPV types, while in a Polish study, these were HPV 42 (2.3%), HPV 66 (1.0%) and HPV 83 (0.9%) [43]. In a representative sample of a United States screening population, HPV 62 and HPV 84 (3.3%), as well as HPV 89 and HPV 61 (2.4%), were the most common types [23]. These findings may be of relevance for HPV type monitoring purposes in the population. Additionally, genotyping of HPV may be beneficial as a form of risk stratification in follow-up monitoring i.e. as a triage method for abnormal cytological findings (ASC-US+). However, it is worthy to note that the risk of developing precancer and cancer from ‘moderate’ risk and LR HPV types is low, representing less than 5% of invasive cervical cancer cases [2, 44]. Therefore, genotyping of non-HR types may be excessive and costly, although they may reduce follow-up monitoring in discordant HPV negative but ASC-US + cases.

### Age-specific prevalence

As consistently described in previous research [16, 40, 45, 46], the prevalence of both HR and LR-HPV decreased with increasing age. This pattern is likely a result of behavioural and biological aspects. In terms of behaviour, sexual activity and the number of sexual partners tends to decrease with age [47]. Our cross-sectional comparison includes several birth cohorts, with more recent birth cohorts having, on average, a higher number of lifetime sexual partners than older ones, as observed in other populations [48]. We observed a slight increase in HPV prevalence in the age group 50–59 years. This second peak of HPV positivity may be explained by hormonal and immune system changes, particularly in the cervix, which might affect HPV detection rather than an actual change in sexual activity during this life period [49]. However, based on natural history studies, others argue that immune response and the number of lifetime sexual partners play a role in the second HPV prevalence peak among older women [50–52]. Since cervical cancer rates in older women are higher than previously estimated after hysterectomy rates were considered [53], these unvaccinated age groups need to be considered in future screening efforts.

### Strengths and limitations

The findings from the MARZY study regarding HPV prevalence provide the first population-based data among HPV non-vaccinated women aged 30 years and above in Germany. Our study reported results by HPV type, age group and cytology including prevalence among women previously unscreened and in an older population, contrasting to the majority of previous studies reporting primarily HPV prevalence of younger age groups and based on routinely screened populations. Obtaining these estimates provide a necessary baseline for understanding the mid- and long-term impact of HPV vaccination status on screening, particularly since screening shifts towards primary HPV testing, there will be a co-existence of HPV vaccinated and unvaccinated cohorts.

There are limitations to our results. We rely on cross-sectional reporting of HPV prevalence where it was not possible to determine whether the infection was newly acquired, persistent or reactivated. Women who participated in the study may not reflect the entire eligible population comprehensively, with older women and women with a migration background less likely to participate [54]. Additionally, we did not adjust our analyses for other known confounding factors as these items were not included in the baseline questionnaire, including lifetime number of sexual partners, age at first sexual intercourse, and sexually transmitted infections. Finally, in contrast to women with lower-grade lesions (ASC-US/LSIL) or normal cytology, our findings relating to high-grade lesions (HSIL+) may be limited due to the low number of cases (*n* = 6) detected, two of which were negative for any HPV type. Our results nonetheless capture HPV prevalence and type distribution on an unvaccinated and population-based sample of older women in Germany and shed valuable light on HPV types not covered by available vaccines and their impact on screening efforts.

## Conclusion

As cohorts of vaccinated women become eligible for screening, and changes in screening recommendations shift towards HPV-based screening, knowing the prevalence and distribution of HPV types in non-vaccinated women is necessary for studying effects of vaccination and screening. A considerable share of HR-HPV infections circulating in the population are due to HPV genotypes not covered by the available vaccines, even when taking cross-protection into account. Monitoring vaccinated and unvaccinated women for these non-vaccine but high-risk HPV genotypes is important for understanding the impact of HPV vaccination and screening efforts and identify optimal prevention strategies. These analyses provide important evidence from a population-based sample of women and can be utilised for screening, monitoring and modelling purposes.

## Electronic supplementary material

Below is the link to the electronic supplementary material.

Supplementary Material 1

## Data Availability

The datasets analysed during the current study are available from the corresponding author upon reasonable request.

## Abbreviations

aOR: Adjusted odds ratio
ASC-US: Atypical squamous cells of undetermined significance
CI: Confidence interval
EIA: Enzyme immunoassay
GEP: Good Epidemiological Practice
HSIL: High-grade intraepithelial lesion
HR: High-risk
HPV: Human papillomavirus
IARC: International Agency for Research on Cancer
LSIL: Low-grade intraepithelial lesion
LR: Low-risk
NILM: Negative for intraepithelial lesion malignancy
PCR: Polymerase chain reaction
RLB: Reverse line blot
WHO: World Health Organization
