# Supplementary material for: Population-based age- and type-specific prevalence of human papillomavirus among non-vaccinated women aged 30 years and above in Germany
Source: BMC Infect Dis. 2024 Sep 19;24:1008. doi: 10.1186/s12879-024-09827-7 (PMC11414300; doi:10.1186/s12879-024-09827-7)
Supplement: Supplementary file 1 — Supplementary Material 1 [file 12879_2024_9827_MOESM1_ESM.docx]

**Supplementary material**

Table S1. HR-HPV type frequency and HPV vaccine coverage by specific type (n= 187 HPV infections)

| **HR-HPV type** | **n** | **Rank by frequency** | **Nonavalent vaccine** | **Bivalent and quadravalent vaccine** |
| --- | --- | --- | --- | --- |
| HPV 16 | 71 | 1 | Yes | Yes |
| HPV 56 | 23 | 2 | No | No |
| HPV 52 | 16 | 3 | Yes | No |
| HPV 31 | 15 | 4 | Yes | No* |
| HPV 45 | 13 | 5 | Yes | No* |
| HPV 18 | 13 | 6 | Yes | Yes |
| HPV 33 | 11 | 7 | Yes | No* |
| HPV 39 | 9 | 8 | No | No |
| HPV 58 | 5 | 9 | Yes | No |
| HPV 51 | 4 | 10 | No | No |
| HPV 35 | 4 | 11 | No | No |
| HPV 59 | 3 | 12 | No | No |
| **All** | **187** |  |  |  |
|  |  |  |  |  |
| Number of infections not covered by vaccines | | | N=43 | N=103 |

HPV: human papillomavirus; HR-HPV: high-risk HPV type;
*HPV types 31, 33, and 45 were considered as cross-protection by the bivalent vaccine^1-3^

**References**

1. Kavanagh K, Pollock KG, Cuschieri K, Palmer T, Cameron RL, Watt C, et al. Changes in the prevalence of human papillomavirus following a national bivalent human papillomavirus vaccination programme in Scotland: a 7-year cross-sectional study. Lancet Infec Dis. 2017;17(12):1293-302.

2. Mariz FC, Gray P, Bender N, Eriksson T, Kann H, Apter D, et al. Sustainability of neutralising antibodies induced by bivalent or quadrivalent HPV vaccines and correlation with efficacy: a combined follow-up analysis of data from two randomised, double-blind, multicentre, phase 3 trials. Lancet Infect Dis. 2021;21(10):1458-68.

3. Tsang SH, Sampson JN, Schussler J, Porras C, Wagner S, Boland J, et al. Durability of Cross-Protection by Different Schedules of the Bivalent HPV Vaccine: The CVT Trial. J Natl Cancer Inst. 2020;112(10):1030-7.
